# Supplementary material for: CARTmath—A Mathematical Model of CAR-T Immunotherapy in Preclinical Studies of Hematological Cancers
Source: Cancers (Basel). 2021 Jun 11;13(12):2941. doi: 10.3390/cancers13122941 (PMC8231202; doi:10.3390/cancers13122941)
Supplement: Supplementary file 1 [file cancers-13-02941-s001.zip › cancers-1173765-supplementary.pdf]

cancers

MDPI

Article

CART*math*—A Mathematical Model of CAR-T Immunotherapy in Preclinical Studies of Hematological Cancers

Luciana R. C. Barros [1,*,†], Emanuelle A. Paixão [2,†], Andrea M. P. Valli [3], Gustavo T. Naozuka [2], Artur C. Fassoni [4] and Regina C. Almeida [5]

1 Center for Translational Research in Oncology, Instituto do Câncer do Estado de São Paulo, Hospital das Clínicas da Faculdade de Medicina ds Universidade de São Paulo, São Paulo 01246-000, Brazil
2 Graduate Program, Laboratório Nacional de Computação Científica, Petrópolis 25651-075, Brazil; earantes@lncc.br (E.A.P.); naozuka@lncc.br (G.T.N.)
3 Computer Science Department, Universidade Federal do Espírito Santo, Vitória 29075-910, Brazil; avalli@inf.ufes.br
4 Institute for Mathematics and Computer Science, Universidade Federal de Itajubá, Itajubá 37500-903, Brazil; fassoni@unifei.edu.br
5 Computational Modeling Department, Laboratório Nacional de Computação Científica, Petrópolis 25651-075, Brazil; rcca@lncc.br
* Correspondence: cto.gestor.redcap@hc.fm.usp.br
† These authors contributed equally to this work.

check for updates

Citation: Barros, L.R.C.; Paixão, E.A.; Valli, A.M.P.; Naozuka, G.T.; Fassoni, A.C.; Almeida, R.C. Supplementary Material for CART*math*—A Mathematical Model of CAR-T Immunotherapy in Preclinical Studies of Hematological Cancers. *Cancers* **2021**, *13*, 2941. https://doi.org/10.3390/cancers13122941

Academic Editors: Víctor M. Pérez-García, Lisette de Pillis, Philipp Altrock and Russell Rockne

Received: 22 March 2021
Accepted: 20 May 2021
Published: 11 June 2021

Publisher's Note: MDPI stays neutral with regard to jurisdictional claims in published maps and institutional affiliations.

Copyright: © 2021 by the authors. Licensee MDPI, Basel, Switzerland. This article is an open access article distributed under the terms and conditions of the Creative Commons Attribution (CC BY) license (https://creativecommons.org/licenses/by/4.0/).

This Supplementary Material (SM) is organized as follows. Section SM-1 describes the procedure used for estimating the model parameter values. Section SM-2 shows the data used for calibration, extracted from [1] and [2]. For completeness, Section SM-3 provides a detailed description of the mathematical analysis of model dynamics. Finally, section SM-4 delivers additional analysis of the virtual population for the HDLM-2 scenario.

1. Estimation of the model parameter values

Parameter estimation is performed for each scenario of interest, from the measurements performed in [1] or [2]. We use the Bayesian approach for the parameter inference, allowing us to consider both the uncertainties in the model and the experimental data [3–5]. A detailed tutorial on this approach focusing on tumor growth models is presented in [6]. The basis of the Bayesian calibration is the Bayes' theorem. To present it in the context of the desired estimation, let $\boldsymbol{\theta} = \{\theta_1, \ldots, \theta_k\}$ be the vector of k parameters we want to estimate and $\boldsymbol{d} = \{d_1, \ldots, d_n\}$ the vector of n experimental data. These data may be, for example, the number of tumor cells measured by BLI [7] at n different times. Like every measure, each one is accompanied by uncertainties $\{\sigma_i\}_{i=1}^{n}$, assumed Gaussian. Let $\boldsymbol{\Sigma}$ be the associated covariance matrix. With these definitions, Bayes' theorem states that:

$$p_{post}(\boldsymbol{\theta}|\mathbf{d}) \propto L(\mathbf{d}|\boldsymbol{\theta})p_{prior}(\boldsymbol{\theta}) \,, \tag{1}$$

where

- p_{prior} is the prior probability distribution corresponding to our *a priori* knowledge on $\boldsymbol{\theta}$;
- L is the likelihood function; it is a conditional function that allows updating the knowledge about the parameters from the experimental data $\mathbf{d}$;
- p_{post} is the joint posterior probability distribution, that represents the *a posteriori* knowledge about the parameters after we know the data.

In this way, Bayes' theorem updates the *a priori* knowledge, integrating information from data. The result is the joint posterior probability distribution p_{post} which represents the improvement of knowledge about the values of the parameters. The distributions of each parameter are the marginal distributions, wherein the MLEs are the maximum *a posteriori* estimates.

For the definition of the likelihood function, we assume that the data are independent and that the uncertainties in the measurements follow the Gaussian distribution as mentioned above. Under these conditions, L is given by:

$$L(\mathbf{d}|\boldsymbol{\theta}) = ((2\pi)^n\ |\boldsymbol{\Sigma}|)^{-1/2} \exp\Big(-\frac{1}{2}(\boldsymbol{d}_m - \boldsymbol{d})^t\ \boldsymbol{\Sigma}^{-1}(\boldsymbol{d}_m - \boldsymbol{d})\Big), \quad (2)$$

in which $\boldsymbol{d}_m$ is the vector of the simulated values obtained with the model referring to the experimental measurements using $\boldsymbol{\theta}$. Note that $L(\mathbf{d}|\boldsymbol{\theta})$ provides a measure of the knowledge brought by the data on parameter values for the model. Thus, the joint posterior probability distribution is determined as:

$$p_{post}(\boldsymbol{\theta}|\mathbf{d}) \propto \exp\Big(-\frac{1}{2}(\boldsymbol{d}_m - \boldsymbol{d})^t\ \boldsymbol{\Sigma}^{-1}(\boldsymbol{d}_m - \boldsymbol{d})\Big) p_{prior}(\boldsymbol{\theta})\ . \quad (3)$$

In the experiments performed in this work, we assumed uniform *a priori* distributions in certain ranges. This means that each calibrated parameter follows a uniform *a priori* probability distribution over interval (a, b), denoted by $\mathcal{U}(a, b)$. As the analytical solution of (3) is restricted to particular cases, p_{post} is obtained via Markov Chain Monte Carlo sampling procedures, usually requiring a large number of model simulations. In this work, we used QUESO (*Quantification of Uncertainty for Estimation, Simulation, and Optimization*), a free library for uncertainty quantification [8]. We used a multilevel Monte Carlo method called DRAM, together with the directives available in the QUESO manual and 20,000 samples. Tumor growth parameters (r and b) were calibrated considering the part of the proposed model associated with the growth of the tumor in the absence of immunotherapy, i.e., $dT/dt = rT(1 - bT)$. Likewise, the cytotoxicity of the CAR-T cells parameter (γ) was calibrated considering the model $dT/dt = -\gamma C_T T$. Since in [2] we also have experimental data associated with the mechanism by which tumor cells inhibit effector cells, for RAJI-like tumors (CD19 + lymphoma), we calibrated the parameter α in $\frac{dC_T}{dt} = \phi C_T - \rho C_T + \theta T C_M - \alpha T C_T$. In this case, we fixed all parameters but α considering experimental data for three treatments, expressing or not the IDO enzyme: RAJI-control + CAR-T 19, RAJI-IDO$^+$ + CAR-T 19 and RAJI-IDO$^+$ + CAR-T 19 + 1-MT.

1.1. Parameter inference for the HDLM-2 + CAR-T 123 scenario

First, we extracted data from [1] to estimate parameters r, b, and γ. For r and b, we used data from Figure 4-B in [1], which are shown here in Table 1. As these data represent untreated tumor growth, we calibrated r and b considering only the tumor growth, i.e., using $dT/dt = rT(1 - bT)$. The uniform priors were set as: $r \sim \mathcal{U}(0.01\ ,\ 0.1)$ day^{-1}, $b \sim \mathcal{U}(10^{-13}, 10^{-10})$ cell^{-1}, and $T_0 \sim \mathcal{U}(10^6, 10^8)$ cell. Notice that the tumor initial condition was also estimated due to great uncertainty about this value. The histograms associated with the posterior distributions for r and b are presented in Figures 1a and 1b, together with their MLEs. To estimate γ, we used data from the standard *in vitro* 4-hour chromium-51 release assay data depicted in Figure 3-B from [1] (shown in Table 2). As described in [1], co-cultures of 1×10^6 cancer cells and CAR-T 123 cells in different proportions were generated. After 4 hours, the number of tumor cells remaining alive was evaluated. To adjust these data, we considered only tumor mortality due to the presence of effector cells, which led to $dT/dt = -\gamma C_T T$. The solution of this equation at 4 hours was used to estimate γ with $\gamma \sim \mathcal{U}(10^{-8}, 10^{-5})$ (cell $\cdot$ day)$^{-1}$ as a prior distribution, which yielded γ_{MLE} and the histogram of the posterior distribution shown in Figure 1c.

Now, since experimental data from effector and memory CAR-T cells were not available in [1], all the other parameters were estimated through simulations of the three population model. This means that extensive simulations were performed, fixing r, b, and γ at their MLEs, until finding a set of parameters that can depict the experimental results that indicate a fast decrease of the tumor after treatment and the tumor elimination after the challenge [1]. Although good representations of the desired scenarios were obtained, it is important to remark that parameter estimation in this way is not unique and could be refined on the lights of more informative data.

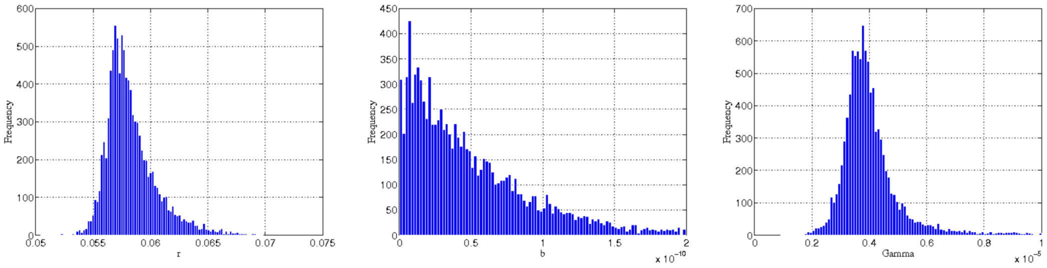

(a) $r_{MLE} = 5.650026 \times 10^{-2}$ day^{-1} **(b)** $b_{MLE} = 1.404029 \times 10^{-12}$ cell^{-1} **(c)** $\gamma_{MLE} = 3.715843 \times 10^{-6}$ (cell $\cdot$ day)$^{-1}$

Figure 1. HDLM-2 + CAR-T 123 scenario: posterior histograms of r (a), b (b), and γ (c) parameters. The maximum *a posteriori* estimates of marginal distributions are indicated by the subscript MLE. Since all priors were assumed uniform distributions, there was a substantial improvement of the knowledge about the parameters with the application of Bayesian inference, with parameters presenting log-normal-like posterior distributions. However, the inference of the inverse of the carrying capacity (b) presents greater uncertainty which may be explained by the lack of saturation of the tumor burden for the untreated mice (see Figure 4B of [1]).

1.2. Parameter inference for the RAJI + CAR-T 19 scenario

RAJI-like tumors (CD19 + lymphoma) expressing or not the IDO enzyme are quite aggressive. None of the available experiments presented in [2] was able to eliminate the tumor, which shows an exponential growth during the seven days corresponding to the monitoring period. Due to this time frame, we set $b = 0$ cell^{-1} in $dT/dt = rT(1 - bT)$.

The r and γ parameters were estimated using the untreated tumor growth model ($dT/dt = rT$) and the tumor decay model due to effector cells ($dT/dt = -\gamma C_T T$), respectively. The uniform priors were set as: $r \sim \mathcal{U}(0.1\ ,\ 0.9)$ day^{-1} and $\gamma \sim \mathcal{U}(10^{-9}, 10^{-7})$ (cel $\cdot$ day)$^{-1}$. The histograms associated with the posterior distributions for r and γ are presented in Figures 2a and 2b, together with their MLEs. Since there is no available data for CAR-T cells (effector or memory), we estimated all the reminder parameters through extensive simulations for the RAJI-control tumor to represent the behavior of the tumor growth depicted in Figure 2C from [2], whose data are shown in Table 3. Once this set of parameters were obtained, we fixed all parameters but α. The α parameter, associated with the mechanism by which tumor cells inhibit effector cells, was calibrated using the prior $\alpha \sim \mathcal{U}(10^{-8}, 3 \times 10^{-8})$ (cell $\cdot$ day)$^{-1}$ in all the following situations. For RAJI-control + CAR-T 19, using the data shown in Table 3, the histogram associated with the posterior distribution for α is presented in Figure 2c, together with its MLE that amounts $\alpha_{MLE} = 1.248506 \times 10^{-8}$ (cell $\cdot$ day)$^{-1}$. For the RAJI-IDO$^+$ tumor, inference of parameter α was performed for the cases with and without 1-MT, using the experimental data presented in Figure 3B from [2] (shown in Table 4). As mentioned before, in these inferences only the parameter α was calibrated to capture the effect of the immunosuppression mechanism, keeping all other parameter values as set in Table 2 (main text). The MLEs obtained for the treatments with RAJI-IDO$^+$ + CAR-T 19 and RAJI-IDO$^+$ + CAR-T 19 + 1-MT are $\alpha_{MLE} = 1.461699 \times 10^{-8}$ (cell $\cdot$ day)$^{-1}$ and $\alpha_{MLE} = 1.261662 \times 10^{-8}$ (cell $\cdot$ day)$^{-1}$, respectively. Note the decrease of α_{MLE} when the IDO inhibitor (1-MT) is used and the similarity between the α_{MLE} values obtained for RAJI-control + CAR-T 19 and RAJI-IDO$^+$ + CAR-T 19 + 1-MT.

2. Data used for inferences

We used data from [1] and [2] for parameter inference. We extracted data from these sources using the free software G3Data Graph Analyzer [9] and organized them in the tables shown below.

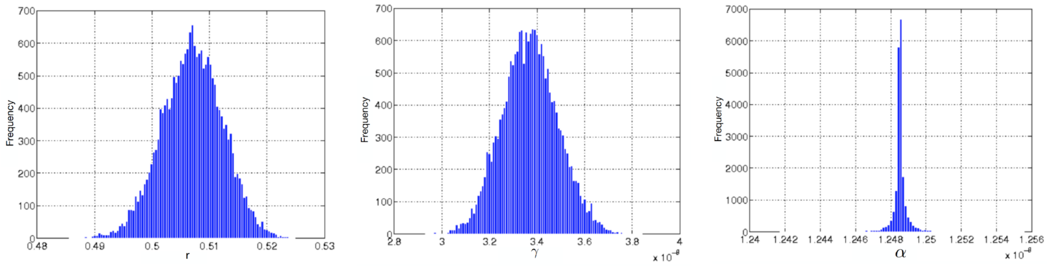

(a) $r_{MLE} = 5.071721 \times 10^{-1} \text{day}^{-1}$ **(b)** $\gamma_{MLE} = 3.365388 \times 10^{-8} (\text{cell} \cdot \text{day})^{-1}$ **(c)** $\alpha_{MLE} = 1.248506 \times 10^{-8} (\text{cell} \cdot \text{day})^{-1}$

Figure 2. RAJI-control + CAR-T 19 scenario: posterior histograms of r (a), γ (b), and α (c) parameters. The maximum *a posteriori* estimates of marginal distributions are indicated by the subscript MLE. As in the case of HDLM-2 cancer, there was a substantial improvement of the knowledge about the parameters with the application of Bayesian inference, with the calibrated parameters also presenting log-normal-like posterior distributions.

Table 1: HDLM-2 tumor burden (data extracted from [1], Figure 4B).

**Day**	**HDLM-2 [BU]** $\rightarrow$ T **[cell]**
48	$9.22 \pm 4.27 \times 10^6$
55	$1.09 \pm 0.36 \times 10^7$
71	$1.01 \pm 0.40 \times 10^8$
85	$2.26 \pm 0.68 \times 10^8$
92	2.02×10^8
97	$9.51 \pm 6.09 \times 10^8$
108	1.31×10^9
112	2.10×10^9
120	1.26×10^9
136	2.73×10^9
142	7.55×10^9

Table 2: Cytotoxic activity against HDLM-2 (data extracted from [1], Figure 3B).

**Effector to target ratio** $C_T : T$	**Live cells** T [cell]
0.3 : 1	2.46×10^6
0.6 : 1	9.90×10^5
1.25 : 1	3.41×10^5
2.5 : 1	1.82×10^5
5 : 1	1.62×10^5
10 : 1	9.65×10^4

Table 3: Tumor burden (data extracted from [2], Figure 2C).

**Day**	**RAJI-IDO$^+$/ RAJI-control [BU]** $\rightarrow$ T **[cell]**
0	$1.05 \pm 0.07 \times 10^8$

**Day**	**RAJI-control + CAR-T 19 [BU]** $\rightarrow$ T **[cell]**
4	$1.6 \pm 2.1 \times 10^8$
7	$5.5 \pm 7.4 \times 10^8$

Table 4: Tumor burden for RAJI-IDO^{+} treated with CAR-T 19 and with CAR-T 19+1-MT (data extracted from [2], Figure 3B).

Day	RAJI-IDO^{+} + CAR-T 19 [BU] $\rightarrow$ T [cell]
4	$4.98 \pm 2.01 \times 10^8$
7	$16.82 \pm 12.71 \times 10^8$

Day	RAJI-IDO^{+} + CAR-T 19 + 1-MT [BU] $\rightarrow$ T [cell]
4	$1.9 \pm 1.3 \times 10^8$
7	$6.9 \pm 4.9 \times 10^8$

Table 5: Cytotoxic activity against RAJI-control and RAJI-IDO^{+} (data extracted from [2], Figure 3D).

Effector to target ratio $C_T : T$	Cytotoxicity [%] $(1 - T\text{ [cell] }/10^6\text{ [cell]})\cdot 100$
40 : 1	73
20 : 1	69
10 : 1	63
5 : 1	56

3. Mathematical analysis of model dynamics

In this section, we present the mathematical analysis for the conditions of existence and stability of equilibrium points in the ODE model. The nondimensional ODE system is given by

$$\frac{dX}{d\tau} = -pX + qZY - sZX\,, \tag{4}$$
$$\frac{dY}{d\tau} = uX - qZY - wY\,, \tag{5}$$
$$\frac{dZ}{d\tau} = Z(1-Z) - XZ\,, \tag{6}$$

where the dimensionless variables are given by $X = \frac{\gamma}{r}C_T$, $Y = \frac{\gamma}{r}C_M$, $Z = bT$, and $\tau = rt$; and the parameters are $p = \frac{\rho-\phi}{r}$, $q = \frac{\theta}{br}$, $s = \frac{\alpha}{br}$, $u = \frac{\epsilon}{r}$, and $w = \frac{\mu}{r}$.

3.1. Equilibrium points

To find the equilibrium points, we set the derivative equal to zero in (6) and find $Z = 0$ or $Z = 1 - X$. Substituting in (4-5) and setting the derivatives to zero, we find the trivial equilibrium $P_0 = (0,0,0)$ for $Z = 0$ and the following system for $Z = 1 - X$:

$$-pX + q(1-X)Y - s(1-X)X = 0\,, \tag{7}$$
$$uX - q(1-Z)Y - wY = 0\,. \tag{8}$$

Note that $X = Y = 0$ is a trivial solution for (7-8) and corresponds to the escape equilibrium point $P_1 = (0,0,1)$. The nontrivial solutions are obtained in the following. Adding (7) and (8), we have

$$(u-p)X - s(1-X)X - wY = 0\,.$$

Solving for Y, we obtain

$$Y = \frac{1}{w}[\vartheta - s(1-X)]X\,, \tag{9}$$

where $\vartheta = u - p > 0$. Substituting (9) in (7) and simplifying, we obtain a second degree polynomial equation:

$$qsX^2 + (q\vartheta - 2qs - sw)X + (pw - q\vartheta + qs + sw) = 0\,. \tag{10}$$

We write (10) as $f(X) = 0$ where $f(X) = a_f X^2 + b_f X + c_f$, and denote the roots as

$$X_2 = \frac{-b_f - \sqrt{b_f^2 - 4a_f c_f}}{2a_f} \quad \text{and} \quad X_3 = \frac{-b_f + \sqrt{b_f^2 - 4a_f c_f}}{2a_f}\,. \tag{11}$$

The solutions of (10) correspond to the nontrivial equilibria P_2 and P_3 given by

$$P_i = (X_i, Y_i, Z_i) = \left(X_i, \frac{[\vartheta - s(1 - X_i)]X_i}{w}, 1 - X_i \right), \qquad i = 2, 3\,.$$

We now analyze the positiveness conditions for equilibria P_2 and P_3. Note that $f(0) = c_f$, $f(1) = pw > 0$ and $f(-\infty) < 0$ since $a_f = qs > 0$. Thus, if $c_f < 0$, analyzing the sign changes of $f(X)$, we conclude that $X_2 < 0 < X_3 < 1$. Note that $c_f < 0$ is equivalent to $\vartheta > \vartheta_T$, where ϑ_T is defined as $\vartheta_T = \frac{(p+s)w}{q} + s$. From (9), also note that $Y_3 > 0$ when $\vartheta > \vartheta_T$, because $\vartheta > s$ and $X_3 \in (0, 1)$. Hence, we have a unique positive equilibrium P_3 when $\vartheta > \vartheta_T$.

For the other case, i.e., for $\vartheta < \vartheta_T$ we make the variable change $Z = 1 - X$ in (9). Note that "$X^* \in [0, 1]$ *is a root of* $f(X) = 0$ *if, and only if,* $Z^* \in [0, 1]$ *is a root of* $f(1 - Z) = 0$". Thus, substituting $X = 1 - Z$ in (9) and simplifying, we obtain

$$sZ^2 + \left(\frac{sw}{q} - \vartheta \right) Z + \frac{pw}{q} = 0\,. \tag{12}$$

We write (12) as $g(Z) = 0$, where $g(Z) = a_g Z^2 + b_g Z + c_g$. Let $\vartheta_0 = \frac{sw}{q}$. If $\vartheta < \vartheta_0$, we have $b_g > 0$. Since $a_g, c_g > 0$, it follows from Descartes's Rule of Signs that we have no positive roots for $\vartheta < \vartheta_0$.

Now, suppose $\vartheta \in (\vartheta_0, \vartheta_T)$. Hence, $b_g < 0$. By Descartes's Rule of Signs, there can be 0 or 2 positive roots. We analyze the discriminant of $g(Z)$, given by

$$\Delta_g = b_g^2 - 4a_g c_g = \vartheta^2 - 2\frac{sw}{q}\vartheta + \frac{sw}{q}\left(\frac{sw}{q} - 4p \right).$$

When $\Delta_g > 0$, $g(Z)$ has two positive roots, and if $\Delta_g < 0$, there are no positive roots. Note that $\Delta_g = \Delta_g(\vartheta)$ is a 2nd degree polynomial in ϑ, with coefficients $a_\Delta = 1$, $b_\Delta = -\frac{2sw}{q}$ and $c_\Delta = \frac{sw}{q}\left(\frac{sw}{q} - 4p \right)$. Thus, the graph of $\Delta_g(\vartheta)$ has a positive concavity and its minimum occurs in

$$\vartheta_V = -\frac{b_\Delta}{2a_\Delta} = \frac{sw}{q} = \vartheta_0\,.$$

Further, note that

$$\Delta_g(\vartheta_V) = -4p\frac{sw}{q} < 0\,.$$

On the other hand, for $\vartheta = \vartheta_T$, we have

$$\Delta_g(\vartheta_T) = \left(s - \frac{pw}{q} \right)^2 > 0\,.$$

Thus, $\Delta_g(\vartheta)$ changes its sign between ϑ_0 and ϑ_T, and it is increasing from ϑ_0. Therefore, if ϑ_{SN} is the root of Δ_g given by

$$\vartheta_{SN} = \frac{-b_\Delta + \sqrt{b_\Delta^2 - 4a_\Delta c_\Delta}}{2},$$

then, we have that $\vartheta_{SN} \in (\vartheta_0, \vartheta_T)$ and that

$$\vartheta_{SN} = \frac{sw}{q} + 2\sqrt{p\frac{sw}{q}}.$$

Thus, if $\vartheta \in (\vartheta_0, \vartheta_{SN})$ then $\Delta_g < 0$ and there are no positive roots; if $\vartheta \in (\vartheta_{SN}, \vartheta_T)$, then $\Delta_g > 0$ and we have two positive roots for $g(Z) = 0$. It remains to analyze whether these positive roots satisfy $Z_i < 1$, in order that $X_i > 0$. We have

$$g(0) = \frac{pw}{q}$$

and

$$g(1) = s + \frac{sw}{q} - \vartheta + \frac{pw}{q} = \vartheta_T - \vartheta > 0, \quad \text{for } \vartheta < \vartheta_T.$$

Hence, g is positive at $Z = 0$ and $Z = 1$. Therefore, either the two roots of g occur in $[0, 1]$ (case A) or occur in $[1, \infty)$ (case B). In case A, the two roots Z_2 and Z_3 give rise to two positive equilibria P_2 and P_3. On the other hand, this does not occur in case B. Visually, we note that what makes the distinction between the two cases is the slope of the graph of $g(Z)$ at $Z = 1$, which is given by

$$g'(1) = 2a_g + b_g = 2s + \frac{sw}{q} - \vartheta.$$

In case A, we have $g'(1) > 0$, that is, $\vartheta < 2s + \frac{sw}{q} = \vartheta_0 + 2s$. In case B, we have $g'(1) < 0$, that is, $\vartheta > 2s + \frac{sw}{q}$. To find the position of $\vartheta_i = \vartheta_0 + 2s$ with respect to ϑ_{SN} and ϑ_T, we note that

$$\vartheta_T = \vartheta_0 + 2m_a,$$

where $m_a = \frac{1}{2}\left(\frac{pw}{q} + s\right)$ is the arithmetic mean between $\frac{pw}{q}$ and s. Then

$$\vartheta_{SN} = \frac{sw}{q} + 2\sqrt{\frac{pw}{q}s} = \vartheta_0 + 2m_g,$$

where $m_g = \sqrt{\frac{pw}{q}s}$ is the geometric mean between $\frac{pw}{q}$ and s. We know that $min\left\{s, \frac{pw}{q}\right\} < m_g < m_a < max\left\{s, \frac{pw}{q}\right\}$. Thus, if $s < \frac{pw}{q}$, we have $s < m_g < m_a$ which implies in $\vartheta_I < \vartheta_{SN} < \vartheta_T$. In this case, if $\vartheta \in (\vartheta_{SN}, \vartheta_T)$ then $\vartheta > \vartheta_I$, and we are in case B; the two roots are outside $[0, 1]$.

Note that, if $s > \frac{pw}{q}$, we have $m_g < m_a < s$ which implies in $\vartheta_{SN} < \vartheta_T < \vartheta_I$. Hence, if $\vartheta \in (\vartheta_{SN}, \vartheta_T)$, then $\vartheta < \vartheta_I$ and we have case A, that is, the roots satisfy $Z_2, Z_3 \in [0, 1]$.

With the above analysis, we identified the existence of three regions in the parameter space where the existence of the nontrivial equilibria P_2 and P_3 is different:

(I) In region $R_1 = \{(\vartheta, s);\ 0 < s \leq \frac{pw}{q},\ \vartheta < \vartheta_T\} \bigcup \{(\vartheta, s);\ s \geq \frac{pw}{q},\ \vartheta < \vartheta_{SN}\}$, P_2 and P_3 are not positive;

(II) In region $R_2 = \{(\vartheta, s);\ s > 0,\ \vartheta > \vartheta_T\}$, only P_3 is positive;

(III) In region $R_3 = \{(\vartheta, s);\ s > \frac{pw}{q},\ \vartheta_{SN} < \vartheta < \vartheta_T\}$, P_2 and P_3 are positive.

3.2. Local stability

The local stability of the model equilibria is determined by assessing the system's Jacobian matrix (J) at each equilibrium point. The Jacobian matrix of the system (4-6) is given by

$$J(X,Y,Z) = \begin{pmatrix} -p-sZ & qZ & qY-sX \\ u & -qZ-w & -qY \\ -Z & 0 & 1-2Z-X \end{pmatrix}. \tag{13}$$

For the trivial equilibrium $P_0 = (0,0,0)$, the eigenvalues of $J(P_0)$ are easily determined, and we find

$$\lambda_1 = -p, \quad \lambda_2 = -w, \quad \text{and} \quad \lambda_3 = 1\,.$$

Therefore, P_0 is always a saddle-point with a two-dimensional stable manifold. It is straightforward to see that the eigenvectors of $J(P_0)$ corresponding to the negative eigenvalues are $v_1 = (1, \frac{u}{-p+w}, 0)$ and $v_2 = (0,1,0)$, and that they generate the XY plane ($Z = 0$), which is invariant with respect to the flow of system (4-6). Thus, the plane $Z = 0$ is the stable manifold of P_0. Due to the invariance property, this means that mathematically the tumor cells cannot be totally eliminated. However, for numerical reasons in our simulations, when the number of tumor cells reaches a number less than 1×10^{-6} (corresponding to threshold $Z < b \times 10^{-6}$), it is set to zero, leading to tumor elimination.

With respect to the escape equilibrium point $P_1 = (0,0,1)$, we have

$$J(P_1) = \begin{pmatrix} -(p+s) & q & 0 \\ u & -(q+w) & 0 \\ -1 & 0 & -1 \end{pmatrix}. \tag{14}$$

The eigenvalues of $J(P_1)$ are $\lambda_1 = -1$ and λ_2 and λ_3, which are eigenvalues of

$$A = \begin{pmatrix} -(p+s) & q \\ u & -(q+w) \end{pmatrix}. \tag{15}$$

We have $tr(A) = -(p+s+q+w) < 0$. On the other hand, $det(A) = q(\vartheta_T - \vartheta)$. Hence, if $\vartheta > \vartheta_T$ then $det(A) < 0$ and P_1 is saddle-point, while if $\vartheta < \vartheta_T$ then $det(A) > 0$ and $P_1 = (0,0,1)$ is locally stable.

Now, we assess the stability of the nontrivial equilibria P_2 and P_3. Using the relation $X_i + Z_i = 1$, we obtain that the Jacobian matrix evaluated at these points is

$$J_i = J(P_i) = \begin{pmatrix} j_{11} & qZ_i & j_{13} \\ u & j_{22} & -qY_i \\ -Z_i & 0 & -Z_i \end{pmatrix}, \tag{16}$$

where

$$j_{11} = -p - sZ_i\,, \quad j_{13} = qY_i - sX_i\,, \quad \text{and} \quad j_{22} = -qZ_i - w\,. \tag{17}$$

Setting dX/dt and $dY/dt = 0$ in (4-5) and comparing with (17), we obtain alternative expressions for j_{11}, j_{13}, and j_{22}, which will also be used:

$$j_{11} = -q\frac{Y_iZ_i}{X_i}\,, \quad j_{13} = p\frac{X_i}{Z_i}\,, \quad \text{and} \quad j_{22} = -u\frac{X_i}{Y_i}\,. \tag{18}$$

From (18), we obtain that

$$j_{11}j_{22} = quZ_i\,. \tag{19}$$

The characteristic polynomial of $J(P_i)$ is

$$\pi(\lambda) = \lambda^3 + a_2\lambda^2 + a_1\lambda + a_0\,,$$

where

$$\begin{aligned} a_2 &= -j_{11} - j_{22} + Z_i\,, \\ a_1 &= j_{11}j_{22} + Z_i(j_{13} - j_{11} - j_{22} - qu)\,, \\ a_0 &= -Z_i(j_{13}j_{22} - j_{11}j_{22} + q(u + qY_i)Z_i)\,. \end{aligned} \tag{20}$$

The Routh–Hurwitz criterion states that the roots of $\pi(\lambda)$ have negative real part if, and only if, $a_2 > 0$, $a_0 > 0$, and $a_2a_1 - a_0 > 0$. Substituting (17) in a_2 given in (20), we obtain

$$a_2 = p + sZ_i + qZ_i + w + Z_i > 0\,, \tag{21}$$

whenever P_i is positive. Thus, the first condition is satisfied for $i = 2,3$. Now, substituting (19) in the expression for a_0 we obtain

$$a_0 = -Z_i\Big(j_{13}j_{22} + q^2Y_iZ_i\Big)\,.$$

Substituting (17), $Z_i = 1 - X_i$ and Y_i as given in (9) and simplifying, we have

$$a_0 = Z_iX_i\Big(2a_fX_i + b_f\Big)\,, \tag{22}$$

where a_f and b_f are the coefficients of (10). By (11), we conclude that

$$a_0(P_2) = -Z_2X_2\sqrt{b_f^2 - 4a_fc_f} \quad \text{and} \quad a_0(P_3) = Z_3X_3\sqrt{b_f^2 - 4a_fc_f}\,.$$

Therefore, if P_2 is a positive equilibrium, then $a_0(P_2) < 0$ and not all eigenvalues of $J(P_2)$ have real negative part. Thus, P_2 is not stable. Since $tr(J_2) < 0$, at least one eigenvalue of P_2 have real negative part. Hence, P_2 is a saddle-point. On the other hand, the second Routh-Hurwitz condition is satisfied for P_3. Let us examine the third condition. First, we simplify the expression for a_1. Substituting (19) in a_1 given in (20), we have

$$a_1 = Z_i(j_{13} - j_{11} - j_{22})\,.$$

Now, substituting (17), then $Z_i = 1 - X_i$ and Y_i from (9) and simplifying, we obtain

$$a_1 = \frac{Z}{w}\Big(a_fX_i^2 + b_fX_i + c_f + (q\vartheta - qs + qw + w^2) + (qs - qw - sw)X\Big)\,,$$

where a_f, b_f, c_f are the coefficients of (10). Since X_i is a root of (10), we have

$$a_1 = \frac{Z_i}{w}\Big(q\vartheta - qs + qw + w^2 + (qs - qw - sw)X\Big)\,. \tag{23}$$

Isolating ϑ in (9), we obtain

$$\vartheta = s(1 - X_i) + w\frac{Y_i}{X_i}\,.$$

Substituting this relation in (23), we obtain

$$a_1 = Z_i\left(w - sX_i + q\left(1 - X_i + \frac{Y_i}{X_i}\right)\right)\,. \tag{24}$$

Now, computing $a_1a_2 - a_0$ using (21), (22), and (24), we obtain

$$\begin{aligned} a_1a_2 - a_0 \quad &= \frac{Y_i}{X_i}(pq + q^2Z_i + qsZ_i + qZ_i) - pqX_i + pq - psX_i + pw - q^2X_iZ_i \\ &+q^2Z_i - q\vartheta X_i - 2qsX_i^2 - 2qsX_iZ_i + 2qsX_i + qsZ_i - qwX_i + qwZ_i \\ &+qw - qX_iZ_i + qZ_i - s^2X_iZ_i + swZ_i - sX_iZ_i + w^2 + wZ_i + qw\frac{Y_i}{X_i}\,. \end{aligned} \tag{25}$$

From $dX/dt = 0$ we have that

$$\frac{Y_i}{X_i} = \frac{p + sZ_i}{qZ_i}\,. \tag{26}$$

From (9), we obtain that

$$\frac{Y_i}{X_i} = \frac{\vartheta + s(1 - X_i)}{w} . \tag{27}$$

Substituting (26) in the first term of (25), and (27) in the last term of (25), and then replacing all X_i by $X_i = 1 - Z_i$, we obtain

$$\begin{aligned} a_1 a_2 - a_0 \quad &= \frac{p^2}{Z_i} + pqZ_i + pq + psZ_i + ps + pw + p + q^2 Z_i^2 + qrZ_i \\ &+ qsZ_i + 2qwZ_i + qZ_i^2 + s^2 Z_i^2 + swZ_i + sZ_i^2 + w^2 + wZ_i . \end{aligned} \tag{28}$$

Equation (28) with $i = 3$ implies that $a_1 a_2 - a_0 > 0$, since $Z_3 > 0$ whenever P_3 is a positive equilibrium. Therefore, we conclude that P_3 is always locally asymptotically stable when it is a positive equilibrium. This concludes the stability analysis.

4. *In silico* experiments with the virtual population for the HDLM-2 scenario

The VP for the HDLM-2 scenario was submitted to the immunotherapy with 1×10^6 CAR-T cells and the overall treatment response was evaluated over 300 days. CR and NR were attained by 4,354 and 645 VM, respectively. Figure 3 shows the tornado plots of the correlation between the variability of the VM parameters with respect to T at $t = 55$ and $t = 75$ days for each of these outcomes. The most critical parameter for both CR and NR cases is the tumor growth rate r, as expected. We can also observe the important role of the cytotoxic coefficient γ in controlling the tumor burden for the CR case, in which its negative effect on T somehow balances the positive effect of r, enabling the success of therapy. While the correlation values at $t = 75$ days decreased when compared to those at $t = 55$ days for the CR cases, they have remained practically the same in NR cases.

We then analyzed the frequency of parameter values in the VP and their distributions for each of the two different therapy outcomes. Figure 4a shows the heterogeneity of the parameter values (normalized) in the VP. Of note, to reflect the variability observed in the experimental data reported in [1], parameters are almost evenly distributed in the range limited by ±60% of the reference values indicated in Table 2 (main text) except ρ, ϵ, and r. Some combinations of these parameters did not generate acceptable VM. For example, the restrictions imposed by the control data of [1] prevented small values of r in the VP, which probably would lead to overall survival outside the range of the actual population.

Figures 4b and 4c show the frequency histograms of the (normalized) parameter values for the two therapy outcomes. CR was more likely to occur for higher differentiation of C_T into C_M (ϵ) and intermediate values of the tumor growth rate (r). On the other hand, the NR cases occurred more often for higher values of the inhibition coefficient of C_T (α) and the tumor growth rate (r), and lower values of the cytotoxic coefficient (γ). Of note, the normalized values of the parameters were evaluated according to their corresponding ranges of ±60% of the reference values.

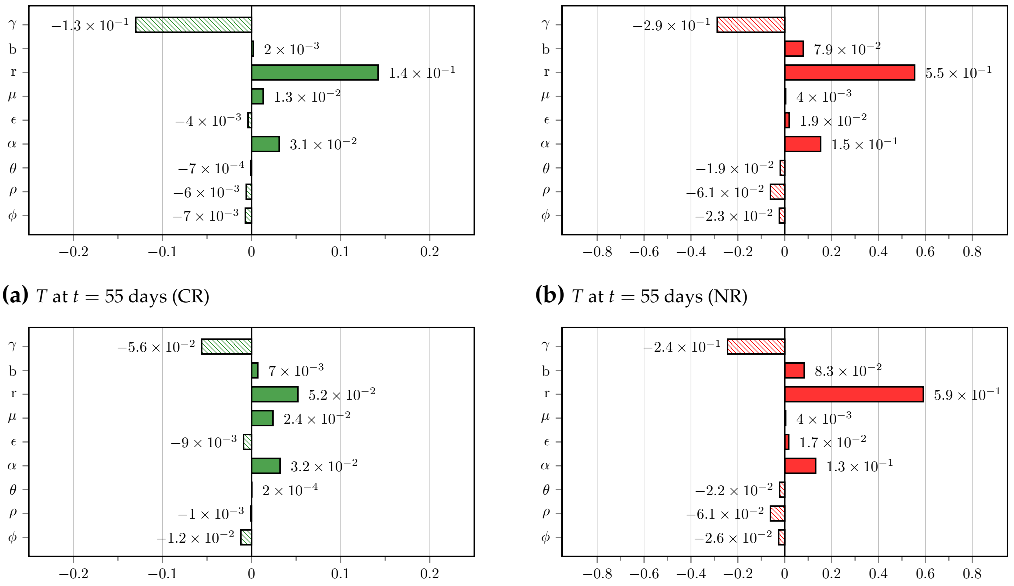

(a) T at $t = 55$ days (CR) **(b)** T at $t = 55$ days (NR)

(c) T at $t = 75$ days (CR) **(d)** T at $t = 75$ days (NR)

Figure 3. Tornado plots of the Pearson correlation with respect to T at $t = 55$ days (top panel) and $t = 75$ days (bottom panel). VP was split into two groups according to the therapy outcomes at 300 *days*: CR (green color) and NR (red color). Solid bars indicate a positive effect while dashed bars indicate a negative one. In all cases, sensitivities with respect to T indicate the critical role of the tumor growth rate r. In the CR case, this positive effect seems to balance the negative effect due to γ.

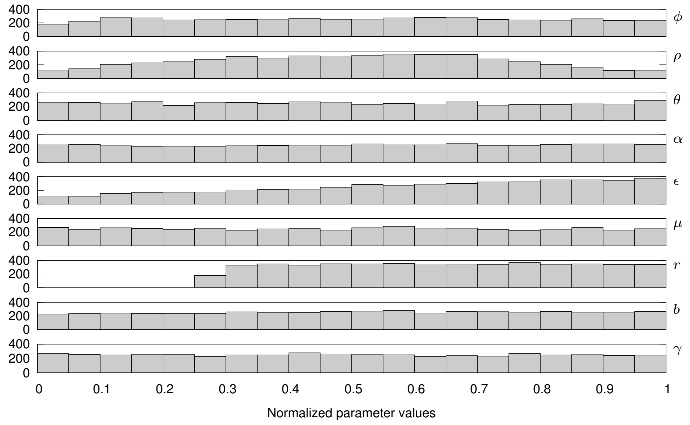

(a) Control

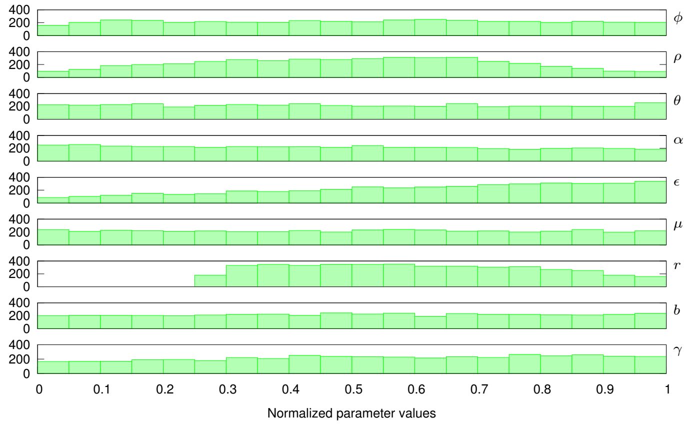

(b) CR

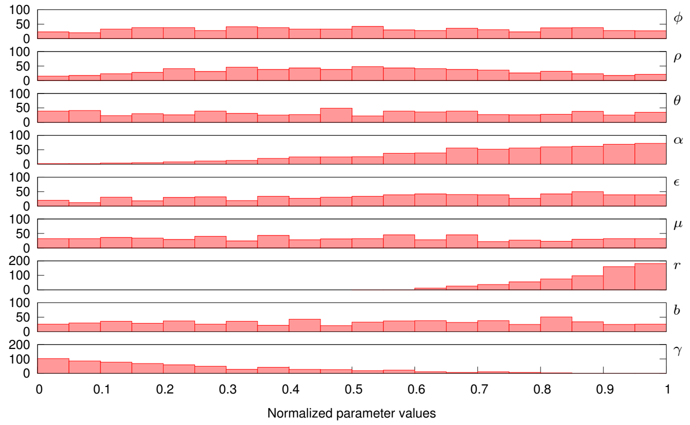

(c) NR

Figure 4. Frequency histograms of the values of the parameters for the VP (a) and different therapy outcomes at $t = 300$ days: (b) CR and (c) NR.

References

1. Ruella, M.; Klichinsky, M.; Kenderian, S.S.; Shestova, O.; Ziober, A.; Kraft, D.O.; Feldman, M.; Wasik, M.A.; June, C.H.; Gill, S. Overcoming the Immunosuppressive Tumor Microenvironment of Hodgkin Lymphoma Using Chimeric Antigen Receptor T Cells. *Cancer Discovery* **2017**, *10*, 1154 – 1167. doi:10.1158/2159-8290.CD-16-0850.
2. Ninomiya, S.; Narala, N.; Huye, L.; Yagyu, S.; Savoldo, B.; Dotti, G.; Heslop, H.E.; Brenner, M.K.; Rooney, C.M.; Ramos, C.A. Tumor indoleamine 2, 3-dioxygenase (IDO) inhibits CD19-CAR T cells and is downregulated by lymphodepleting drugs. *Blood* **2015**, pp. 3905 – 3916. doi:10.1182/blood-2015-01-621474.
3. Oden, J.T.; Hawkins, A.; Prudhomme, S. General diffuse-interface theories and an approach to predictive tumor growth modeling. *Mathematical Models and Methods in Applied Sciences* **2010**, *20*, 477 – 517. doi:10.1142/S0218202510004313.
4. Tarantola, A. *Inverse problem theory and methods for model parameter estimation*; SIAM: Philadelphia, 2005.
5. Muehleisen, R.T.; Bergerson, J. Bayesian Calibration - What, Why And How. International High Performance Buildings Conference, 2016, p. Paper 167.
6. Collis, J.; Connor, A.J.; Paczkowski, M.; Kannan, P.; Pitt-Francis, J.; Byrne, H.M.; Hubbard, M.E. Bayesian Calibration, Validation and Uncertainty Quantification for Predictive Modelling of Tumour Growth: A Tutorial. *Bulletin of Mathematical Biology* **2017**, *79*, 939 – 974. doi:10.1007/s11538-017-0258-5.
7. Allen, A.B.; Gazit, Z.; Su, S.; Stevens, H.Y.; Guldberg, R.E. In vivo bioluminescent tracking of mesenchymal stem cells within large hydrogel constructs. *Tissue Engineering: Part C* **2012**, *20*, 806 – 816. doi:10.1089/ten.TEC.2013.0587.
8. Prudencio, E.E.; Schulz, K.W. The parallel C++ statistical library 'QUESO': Quantification of Uncertainty for Estimation, Simulation and Optimization. Euro-Par 2011: Parallel Processing Workshops; Springer: Bordeaux, France, 2012; pp. 398 – 407.
9. G3Data. G3Data Graph Analyzer, 2011. Version 1.5.4. Available online: https://github.com/pn2200/g3data (accessed on 13 July 2018).
